# Protective Effects of Fucoxanthin against Alcoholic Liver Injury by Activation of Nrf2-Mediated Antioxidant Defense and Inhibition of TLR4-Mediated Inflammation

**DOI:** 10.3390/md17100552

**Published:** 2019-09-27

**Authors:** Jiawen Zheng, Xiaoxiao Tian, Wen Zhang, Pingan Zheng, Fangfang Huang, Guofang Ding, Zuisu Yang

**Affiliations:** 1 Zhejiang Provincial Engineering Technology Research Center of Marine Biomedical Products, School of Food and Pharmacy, Zhejiang Ocean University, Zhoushan 316022, China; jwzheng1996@163.com (J.Z.); TIANXIAOXIAO0208@163.com (X.T.); zhangwen1225z@163.com (W.Z.); dinggf2007@163.com (G.D.); 2 Zhejiang Hailisheng Group Co., Ltd., Zhoushan 316021, China; zhengpingan@hailisheng.com

**Keywords:** alcoholic liver injury, fucoxanthin, oxidative stress, Nrf2, TLR4

## Abstract

Fucoxanthin (Fx) is a natural extract from marine seaweed that has strong antioxidant activity and a variety of other bioactive effects. This study elucidated the protective mechanism of Fx on alcoholic liver injury. Administration of Fx was associated with lower pathological effects in liver tissue and lower serum marker concentrations for liver damage induced by alcohol. Fx also alleviated oxidative stress, and lowered the level of oxides and inflammation in liver tissue. Results indicate that Fx attenuated alcohol-induced oxidative lesions and inflammatory responses by activating the nuclear factor erythrocyte-2-related factor 2 (Nrf2)-mediated signaling pathway and down-regulating the expression of the toll-like receptor 4 (TLR4)-mediated nuclear factor-kappa B (NF-κB) signaling pathway, respectively. Our findings suggest that Fx can be developed as a potential nutraceutical for preventing alcohol-induced liver injury in the future.

## 1. Introduction

The liver is the main organ used for alcohol metabolism; alcohol damages liver cells, which can lead to alcoholic liver disease (ALD) [1]. Acute ALD refers to a disease caused by liver damage associated with heavy drinking over a short period [2,3]. ALD is a worldwide public health problem; its prevalence and morbidity have increased each year as a consequence of increased alcohol abuse rates, which damages human physical health [4,5]. Excessive drinking can cause liver damage to varying degrees, especially in the short term; a large amount of alcohol abuse, for instance, can cause severe liver damage [1,6]. Protective methods are one way of counteracting the rising rates of ALD, which may be possible by utilizing the properties of natural active substances, rather than traditional drugs. 

Large amounts of alcohol in the body can be dehydrogenated into acetaldehyde and acetate under the catalysis of alcohol dehydrogenase (ADH) and acetaldehyde dehydrogenase (ALDH) [7]. Acetaldehyde can damage mitochondria and inhibit the tricarboxylic acid cycle; it also reacts with intracellular macromolecules such as proteins and lipids to form complexes. Acetaldehyde production and the release of adrenaline as a result of ethanol can also cause hepatic vasoconstriction, elevated intrahepatic sinus pressure, or hypoxia of liver tissue, which can lead to vacuolar degeneration of hepatocytes [8,9,10]. Alcohol metabolism also produces a large number of harmful free radicals, affecting the activities of antioxidant enzymes, especially glutathione levels in cells. The body’s antioxidant capacity is insufficient to cope with the accumulation of free radicals, which can lead to the accumulation of lipid peroxides, causing damage to liver cells [11,12,13]. Furthermore, mass alcohol consumption stimulates the production of endotoxin (LPS) in the body, activates liver macrophages, namely Kupffer cells, and leads to high expression of the toll-like receptor 4 (TLR4), which in turn releases a large concentration of reactive oxygen species (ROS) and tumor necrosis factor (TNF-α) or other inflammatory factors that can accelerate downstream inflammation and oxidative damage [14,15,16,17]. When LPS enters the liver this directly damages hepatocytes and promotes the inflammatory cells to produce a large number of inflammatory mediators, which can induce inflammatory cell infiltration and hepatocyte necrosis [18]. Studies have shown that LPS and TNF-α can directly or indirectly exert toxic effects on liver cells [19]. At present, the exact etiopathogenesis of alcoholic liver damage has not been fully clarified which is, therefore, one of the hotspots of current research. 

**Figure 1:**
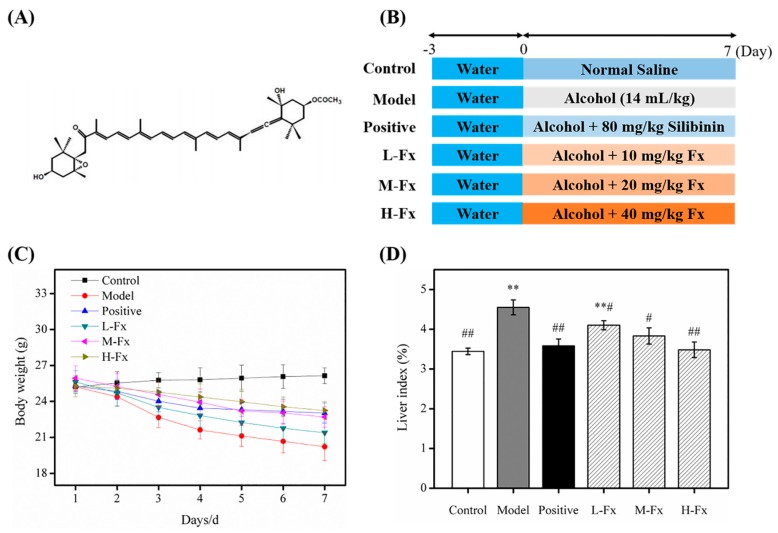
Effect of fucoxanthin (Fx) on bodyweight and liver index in mice with alcoholic liver injury. (**A**) The chemical structure of Fx. (**B**) The experimental design in 7 days. (**C**) The bodyweight of mice. (**D**) The liver index of mice. Data are given as mean ± standard deviation (SD) (*n* = 8). ** *p* < 0.01 vs. Control group, ^#^ *p* < 0.05, ^##^ *p* < 0.01 vs. Model group.

The ocean accounts for 70% of the Earth’s surface area and is an important marine life support system as part of the biological world [20]. The multiplicity of the ocean environment has led to marine organisms that are diverse and widely distributed. Since the marine environment is completely different to terrestrial environments, many marine organisms produce active substances with specific functions and special structures: many of which have antibacterial, antiviral and antitumor activities [21]. In recent years, in-depth research has been conducted on marine resources, which has given a new outlook for drug development. Recent studies have reported the effects of natural extracts on the prevention or improvement of alcoholic liver damage, for example, astaxanthin [22] and aplysin [23] have shown protective effects on alcoholic liver damage in vivo. Fucoxanthin (Fx) is a red-orange carotenoid extracted from natural seaweed [24]. The chemical structure of Fx is shown in Figure 1A. Fx has been reported to have strong antioxidative effects, and has other biological activities, such as anti-obesity, anti-inflammatory and anti-cancer properties [25,26,27]. In addition, Fx was shown to improve glycolipid metabolism in type 2 diabetic mice and improve ventricular rhythm and muscle function models in aging mice [28]. Nevertheless, the role of Fx in alcohol-induced liver injury has not been reported. Our current research aims to investigate the prophylactic function of Fx on acute ALD by monitoring oxidative stress and inflammation. The potential mechanism of Fx was explored by observing oxidative stress signaling pathways and inflammatory signaling pathways in mice. These studies will help to elucidate the latent protective mechanisms of Fx for acute alcoholic liver injury.

## 2. Results and Discussion

### 2.1. Effects of Fucoxanthin (Fx) on Bodyweight and Relative Liver Weight

Mice were weighed each day during the experimental period; changes in the weight are shown in Figure 1C. After 7 days of treatment, mice given alcohol had significantly lower bodyweights than control mice. The bodyweight of H-Fx group mice was much greater than that of Model group mice. Regarding the relative liver weight (Figure 1D), except for the L-Fx group, there was no obvious difference between the Positive group, Fx groups and Control group, but the liver weight of these groups was significantly lower than those of the Model group, indicating that Fx may have a protective effect on liver tissue.

### 2.2. Effects of Fx on Alcohol-Induced Liver and Stomach Injury

**Figure 2:**
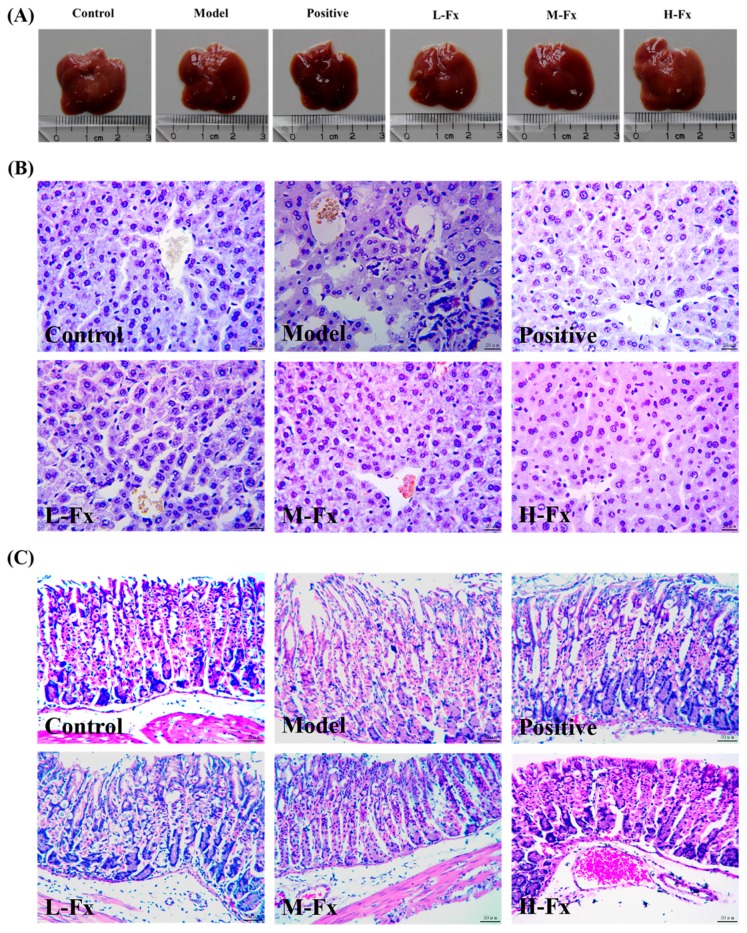
Effect of Fx on alcohol-induced liver and stomach injury. (**A**) Macroscopic picture of livers; (**B**) hematoxylin and eosin (H&E) stained liver tissues. (400× magnification); (**C**) H&E stained stomach tissues. (200× magnification).

Changes in liver and stomach tissue were evaluated by histopathological observation and hematoxylin and eosin (H&E) staining to assess the protective effects of Fx on these tissues. Each group of liver specimens is shown in Figure 2A. Macro-observation showed that the Model group had an enlarged liver with a rough surface and plaque degeneration. However, administration of Fx and Silibinin modified the degree of swelling and degeneration of liver induced by alcohol.

As shown in Figure 2B, H&E staining further confirmed that Fx inhibits alcohol-induced liver injury. The hepatic lobule was structurally intact, the hepatic cord was arranged radially around the central vein, and the hepatic sinus was clear in the Control group. In contrast, in the Model group, the hepatic lobule structure was disordered and incomplete, the hepatic cord arrangement was disordered, and some hepatocytes were necrotic. In contrast to the Model group, the Fx groups had obvious signs of repair; the low, medium, and high doses were associated with a trend of gradual repair. In the H-Fx group, hepatic lobules were structurally intact and hepatic cords were neatly arranged; the hepatic sinus gap was clear and no vacuoles were observed. The signs of repair in the H-Fx group was more obvious than those in the Positive group. The results were consistent with results from Liu et al. [29], who found that astaxanthin can prevent alcoholic fatty liver disease by modulating mouse gut microbiota, indicating that Fx could possess a protective effect on alcohol-induced liver injury in vivo.

H&E staining results for stomach tissue are shown in Figure 2C. The epithelial and lamina propria cells were arranged closely and neatly in the Control group, and the main cells and parietal cells were normal. However, large areas of epithelium were exfoliated in the Model group, the structures of main cells and parietal cells were destroyed, their arrangement was disordered, and local necrosis was observed. There more signs of greater cellular health in Positive and Fx groups than in the Model group, such as closer arrangement of cells and clearer cell structures. This indicates that Fx has a protective effect on the gastric mucosa.

### 2.3. Effects of Fx on Serum Aspartate Transaminase (AST) and Alanine Transaminase (ALT) Activities

**Figure 3:**
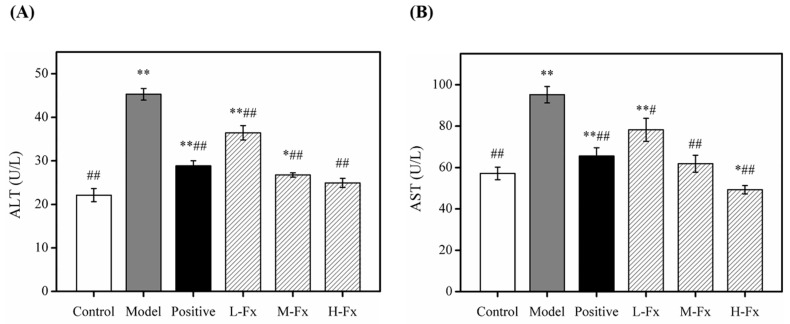
Effect of Fx on serum level of ALT (**A**) and AST (**B**) activities in mice with alcoholic liver injury. Data are given as mean ± SD (*n* = 8). * *p* < 0.05, ** *p* < 0.01 vs. Control group, ^#^ *p* < 0.05, ^##^ *p* < 0.01 vs. Model group.

Aspartate transaminase (AST) and alanine transaminase (ALT) are soluble enzymes present in hepatocyte cytoplasm [15]. After hepatocyte injury, the permeability of the cell membrane increases, causing ALT and AST to enter the bloodstream. Therefore, serum ALT and AST activities can reflect the degree of hepatocyte damage. Serum ALT and AST activities are conventionally used as sensitive markers to evaluate liver damage [30,31]. The ALT and AST activities recorded from Model group mice were significantly greater than those from Control group mice, indicating that Model group mice were successfully established as an alcoholic liver injury model (Figure 3). ALT and AST activities of Positive group and Fx group mice were significantly less than those of the Model group and gradually tended to Control group (Figure 3). These results indicate that Fx could control ALT and AST activities, thereby reducing alcohol-induced liver injury; this hypothesis is also supported by findings by Han et al. [32].

### 2.4. Effects of Fx on Serum and Hepatic Level of Triglyceride (TG)

**Figure 4:**
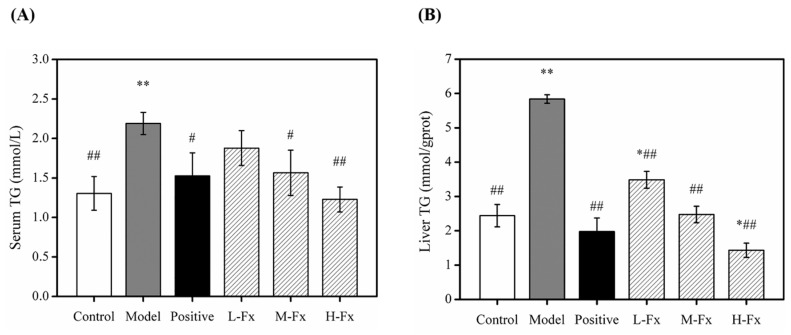
Effects of Fx on serum level of triglyceride (TG) (**A**) and hepatic level of TG (**B**) in mice with alcoholic liver injury. Data are given as mean ± SD (*n* = 8). * *p* < 0.05, ** *p* < 0.01 vs. Control group, ^#^ *p* < 0.05, ^##^ *p* < 0.01 vs. Model group.

Heavy drinking can lead to accumulation of lipids in the liver. During metabolic processing of alcohol, large amounts of lipase are released, which promotes the synthesis of triglyceride (TG) and causes fat to deposit in liver cells. If a fatty lesion develops in liver tissue, blood lipid levels can rise [33]. To further illustrate the effect of Fx pretreatment on lipid metabolism in alcohol-induced mice, serum TG levels (A) hepatic TG levels (B) were measured, as shown in Figure 4. TG levels in the serum and liver of Model group mice increased significantly compared to the Control group, indicating that alcohol caused lipid accumulation, resulting in the disorder of lipid metabolism and liver damage. The TG levels in serum and liver were significantly less in Positive and Fx groups than Model group. These results indicate that Fx can effectively alleviate alcohol-induced liver fat deposition.

### 2.5. Effects of Fx on Hepatic Levels of Malonaldehyde (MDA) and Antioxidant Enzymes

Alcohol-induced oxidative stress is a vital element in the nosogenesis of acute ALD [34]. Metabolism of alcohol can generate high levels of ROS, which can affect the body’s antioxidant systems, increasing the formation of products that cause oxidative stress; this can increase the level of oxidative stress in many tissues, especially those of the liver [35,36]. Antioxidants protect cells from ROS, but antioxidant activity is easily quenched by excess lipid peroxides, which can lead to liver damage [37,38]. Table 1 shows the effect of Fx on liver total antioxidant capacity (T-AOC), and malonaldehyde (MDA), glutathione peroxidase (GSH-Px), superoxide dismutase (SOD) and catalase (CAT) content in mice. The activities of T-AOC, GSH-Px, SOD, and CAT in the Model group were significantly lower than those of the Control group, but the MDA content was significantly higher. The activities of T-AOC, GSH-Px, SOD and CAT measured for Positive and the Fx groups were significantly higher than those of the Model group, and the MDA content was significantly lower. GSH-Px and SOD have a crucial effect on the balance between oxidation and antioxidant capacity in vivo, suggesting that alcohol significantly increased the peroxide levels in the liver and decreased the liver’s antioxidant capacity [39]. Therefore, MDA may be a product of free radicals acting on lipid peroxidation. The degree of lipid peroxidation in the body can be reflected by MDA content, which is a common indicator of membrane lipid peroxidation [40]. This indicates that Fx may effectively prevent the excessive oxidation of mouse hepatocytes caused by alcohol intake, and could enhance the body’s antioxidant capacity, greatly protecting against alcoholic liver injury. Qu et al. [41] reported that ginsenoside Rk3 protected against alcohol-induced liver injury in mice, which is consistent with the results of this study.

### 2.6. Effects of Fx on Hepatic Alcohol Dehydrogenase (ADH) and Acetaldehyde Dehydrogenase (ALDH) Activities

**Figure 5:**
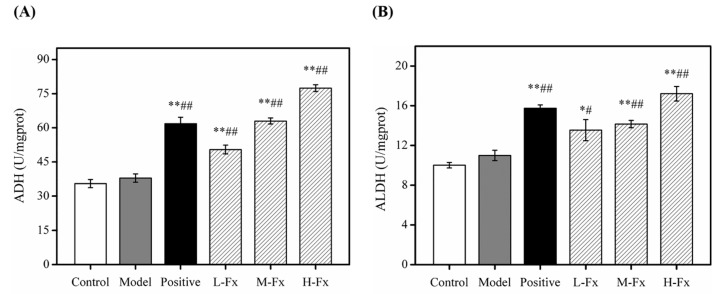
Effects of Fx on hepatic alcohol dehydrogenase (ADH) (**A**) and acetaldehyde dehydrogenase (ALDH) (**B**) activities in mice with alcoholic liver injury. Data are given as mean ± SD (*n* = 8). * *p* < 0.05, ** *p* < 0.01 vs. Control group, ^#^ *p* < 0.05, ^##^ *p* < 0.01 vs. Model group.

The liver is the main organ used in alcohol metabolism, ADH and ALDH are important enzymes for this process [42]. When the body ingests a large amount of alcohol, it is dehydrogenated through to form acetaldehyde via ADH, then converted to acetic acid by the action of ALDH; finally, the acetic acid metabolized to water and CO_2_ via the tricarboxylic acid cycle. ADH and ALDH can metabolize 80% of liver ethanol, which has important anti-alcoholic activity. By detecting the viability of ADH and ALDH, it is possible to reflect the anti-alcoholic capacity of hepatocytes, and further evaluate the protection of Fx on the liver [8,43]. As shown in Figure 5, after alcohol was administered to mice, hepatic ADH (A) and ALDH (B) activities in the Model group increased slightly, but these activities were not significantly different to those of Control group. However, ADH and ALDH activities of groups that had Fx and Silibinin administered were significantly greater than those of Control group. This indicates that Fx has a certain anti-alcoholic effect, thereby preventing alcohol-induced liver damage.

### 2.7. Effects of Fx on the Levels of Pro-Inflammatory Cytokines in Liver Tissue

**Figure 6:**
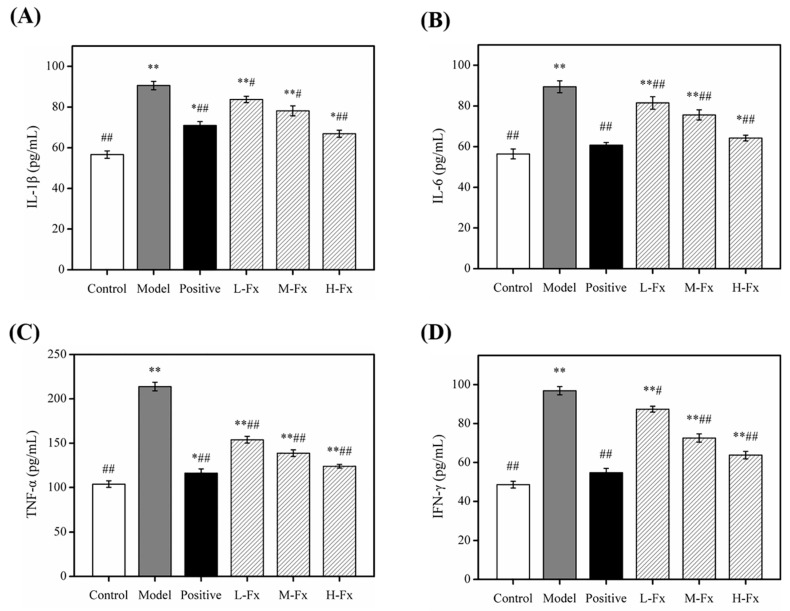
Effects of Fx on the levels of pro-inflammatory cytokines in liver. The release of interleukin-1β (IL-1β) (**A**), interleukin-6 (IL-6) (**B**), tumor necrosis factor (TNF-α) (**C**) and interferon-γ (IFN-γ) (**D**). Data are given as mean ± SD (*n* = 8). * *p* < 0.05, ** *p* < 0.01 vs. Control group, ^#^ *p* < 0.05, ^##^ *p* < 0.01 vs. Model group.

After long-term or heavy drinking, the excessive accumulation of lipids caused by alcohol intake may cause direct or indirect damage to liver cells, produce inflammatory responses, increase the expression of pro-inflammatory factors, and lead to alcoholic liver inflammation. The decomposition of a large amount of alcohol also stimulates the immune functioning of liver cells and activates Kupffer cells [44,45]. Activated Kupffer cells rapidly activate transcriptional regulators such as nuclear factor-kappa B (NF-κB), producing large amounts of inflammatory factors such as TNF-α, interleukin-1β (IL-1β), interleukin-6 (IL-6), interferon-γ (IFN-γ), and inflammatory mediators; this can aggravate the inflammatory infiltration and injury of liver tissue, eventually leading to liver inflammation and hepatocyte necrosis and apoptosis [46,47]. To investigate whether Fx can reduce the inflammatory response caused by alcohol-induced liver injury, we tested the expression of related inflammatory factors. After alcohol induction, levels of 4 cytokines released in liver tissue had significantly increased (Figure 6), indicating that these 4 pro-inflammatory factors participated in the process of alcohol-induced liver injury in mice. The expression of various factors in liver tissue was inhibited by different degrees in mice administered with silibinin and Fx. This indicates that Fx can effectively control the secretion of pro-inflammatory factors, thereby reducing the inflammatory response caused by the alcohol-induced liver injury.

### 2.8. Effects of Fx on Nuclear Factor Erythrocyte-2-Related Factor 2 (Nrf2)-Mediated Antioxidant Response

**Figure 7:**
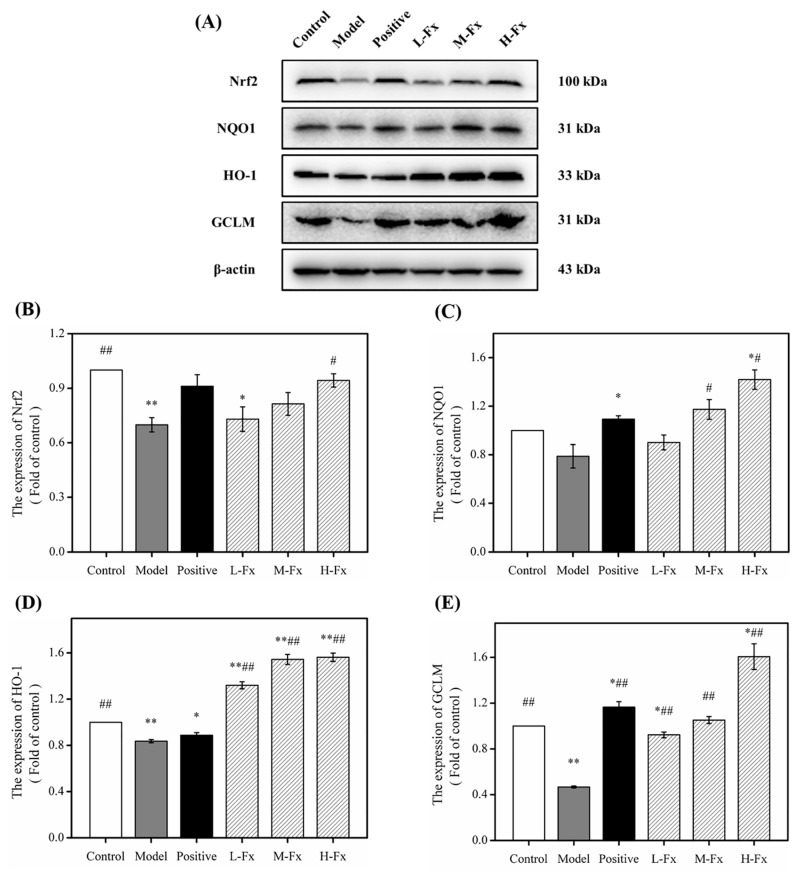
Effect of Fx on the nuclear factor erythrocyte-2-related factor 2 (Nrf2)-mediated antioxidant response. (**A**) Western blot analysis of Nrf2, NQO1, HO-1 and GCLM in livers; (**B**) quantitative analysis for Nrf2; (**C**) Quantitative analysis for NQO1; (**D**) quantitative analysis for HO-1; (**E**) quantitative analysis for GCLM. Data are given as mean ± SD (*n* = 8). * *p* < 0.05, ** *p* < 0.01 vs. Control group, ^##^ *p* < 0.01 vs. Model group.

To ulteriorly investigate the antioxidant mechanism of Fx on alcohol-induced liver injury, the nuclear factor erythrocyte-2-related factor 2 (Nrf2) content of cells and its downstream proteins were detected by Western blot. Nrf2 is a crucial regulatory transcription factor that acts upstream of the antioxidant defense system and plays a major part in regulating redox equilibrium [48]. Phosphorylation occurs when Nrf2 is stimulated by free radicals or nucleophiles; Nrf2 and cytoskeleton-related proteins then dissociate and Nrf2 enters the nucleus to initiate the expression of antioxidant enzymes such as nicotinamide quinone oxidoreductase 1 (NQO1), heme oxygenase-1 (HO-1), and glutamate-cysteine ligase modifier subunit (GCLM). *NQO1*, *HO-1* and *GCLM* are downstream target genes of Nrf2 that function as antioxidant molecules [49,50,51]. Studies have shown that a large amount of alcohol can inhibit the normal activation of Nrf2 expression, leading to an increase in alcohol-induced oxidative stress [52]. As shown in Figure 7, alcohol-induced liver injury can significantly reduce the levels of Nrf2 protein, further decreasing the content of downstream antioxidant protein regulated by Nrf2, and the expression of downstream proteins NQO1, HO-1 and GCLM was significantly reduced, indicating that alcoholic liver injury can significantly inhibit the Nrf2 signaling pathway. However, Positive and Fx groups had significantly greater levels of these proteins than Model groups. Fx was associated with an increase in the levels of Nrf2 protein and its downstream target proteins in a dose-dependent manner. The results indicate that Fx minimizes the damage caused by alcohol to the liver of mice by activating Nrf2-mediated antioxidant responses.

### 2.9. Effect of Fx on Toll-Like Receptor 4 (TLR4)-Induced Inflammatory Response

**Figure 8:**
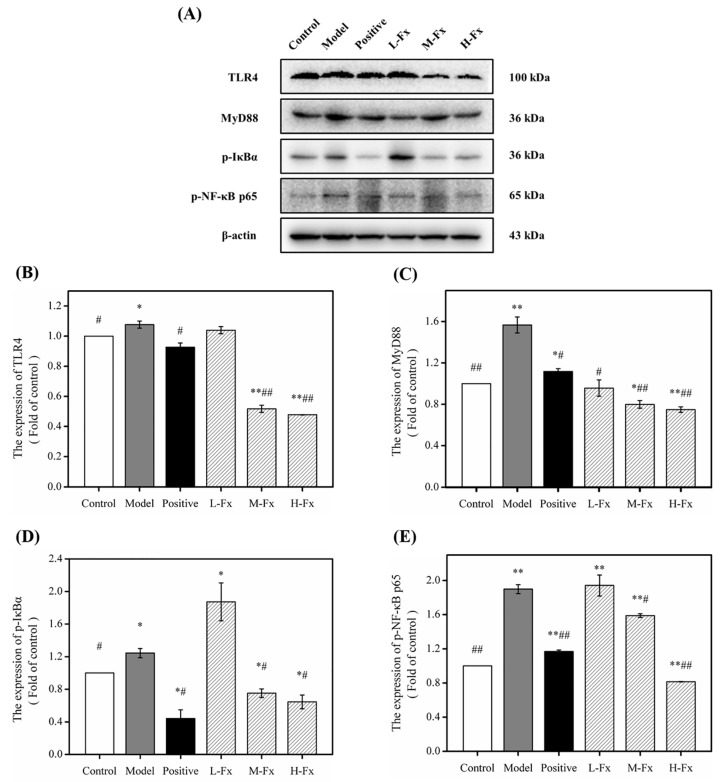
Effect of Fx on toll-like receptor 4 (TLR4)-induced inflammatory response. (**A**) Western blot analysis of TLR4, myeloid differentiation factor 88 (MyD88), p-IκBα and p-NF-κB p65; (**B**) the protein levels of TLR4; (**C**) the protein levels of MyD88; (**D**) the protein levels of p-IκBα; (**E**) the protein levels of p-NF-κB p65. Data are given as mean ± SD (*n* = 8). * *p* < 0.05, ** *p* < 0.01 vs. Control group, ^#^ *p* < 0.05, ^##^ *p* < 0.01 vs. Model group.

TLR-induced signaling pathways are the main pathway leading to inflammatory responses in alcohol-induced liver injury [46]. TLR4 is a specific exogenous receptor of LPS; after ingesting a large amount of alcohol, TLR4 converts to form LPS activated TLR4, creating downstream signals, and activating transcription factors through intracellular signaling pathways to promote inflammation [53,54]. Myeloid differentiation factor 88 (MyD88) is an important adaptor protein molecule of TLR4, that can be activated by TLR4. Activated MyD88 can activate downstream NF-κB and mediate the release of inflammatory factors [53]. NF-κB as the most important transcriptional regulator in the LPS/TLR4 inflammatory signal transduction pathway, and plays a key regulatory role in the transcriptional synthesis of the pathway’s inflammatory mediators [55,56]. Under normal conditions, NF-κB forms a complex with its inhibitor IκB in an inactive state, which causes the phosphorylation and degradation of IκB. NF-κB is then activated and enters the nucleus, which then leads to the transcription and release of inflammatory cytokines. To investigate the anti-inflammatory mechanism of Fx on the protective effects of alcohol-induced liver injury, Western blots were used to analyze the concentrations of TLR4-induced signaling pathway-related proteins. As shown in Figure 8, after alcohol induction, the concentrations of TLR4 were significantly increased, and a downstream signal cascade was initiated, leading to significant up-regulation of MyD88, p-IκBα and p-NF-κBp65. However, Fx pretreatment groups had significantly greater concentrations of downstream proteins than the Control group after alcohol administration. Overall, results indicate that Fx can attenuate alcohol-induced hepatic inflammatory responses by inhibiting TLR4-induced signaling pathways.

## 3. Materials and Methods 

### 3.1. Materials and Chemicals 

Fucoxanthin was purchased from Shandong Jiejing Group Corporation (Rizhao, Shandong, China). Silibinin (powdered capsule) was purchased from Tianshili Shengte Pharmaceutical Co., Ltd. (Tianjin, China). Enzyme-linked immunosorbent assay (ELISA) kits and bicinchoninic acid (BCA) protein assay kit were purchased from BOSTER (Wuhan, China). AST, ALT, TG, T-AOC, MDA, GSH-Px, SOD, CAT, ADH and ALDH kits were all obtained from the Jiancheng Bioengineering Institute (Nanjing, China). Antibodies against Nrf-2, NQO1, TLR4 were obtained from Affinity Biosciences, Inc. (Cincinnati, OH, USA). Antibodies against MyD88 were purchased from BOSTER (Wuhan, China). Antibodies against HO-1, p-IκBα, GCLM and p-NF-κB p65 were obtained from Proteintech Group, Inc. (Princeton, NJ, USA). All reagents were of analytical grade.

### 3.2. Animals and Treatment

Male ICR mice (6 weeks old) were purchased from the Experiment Animal Center of Zhejiang Province (Hangzhou, China). The protocol was approved by the Experimental Animal Ethics Committee of Zhejiang Ocean University (Zhoushan, China), and our animal certificate was No. SCXK (ZHE 2014-0001). Mice were housed in a constant condition of temperature (22 ± 2 °C) and humidity (55 ± 5%) on a 12-h light/dark cycle. After 7 days of adaptive feeding, 48 mice were stochastically allocated into 6 groups: control group mice were given normal saline (Control), model group mice were given alcohol by gavage (56% *w/v*, total 10 g/kg b.w.) twice a day, separated by half an hour (Model), positive group mice were given alcohol by gavage 80 mg/kg b.w. Silibinin was used on mice in the positive control group (Positive) [57], and for the different Fx groups, mice were given alcohol supplemented with Fx by oral administration of 10 (L-Fx), 20 (M-Fx), and 40 (H-Fx) mg/kg b.w. Positive group and Fx group mice were given alcohol after taking the drug for half an hour. The experimental procedure is shown in Figure 1B and the whole experiment lasted for 7 days. All mice were given controlled food allowances but they could drink water at will. Mouse weights were recorded each day. At day 7, mice were fasted after 12 hours and euthanized by cervical dislocation; serum and tissues were stored at −80 °C. 

### 3.3. Histopathological Analysis

Histopathological alteration of the liver and stomach tissues were measured by H&E staining using a standard procedure [58]. For H&E staining, tissue from the isolated stomach and the left lobe of the liver were fixed in 4% neutral-buffered formalin solution between 24 h to 48 h, dehydrated stepwise with gradient alcohol, clarified using xylene, then embedded in paraffin. The tissue was then cut into 4 μm thick pieces using a microtome (Leica RM2135, Leica Instruments GmbH, Wetzlar, Germany) for H&E staining. Photomicrographs were observed under an optical microscope (Biological microscope CX31, Olympus, Tokyo, Japan) and photographed at 200× or 400× magnification.

### 3.4. Serum Biochemical Analysis

The retro-orbital blood samples were centrifuged (10,000 rpm) at 4 °C for 5 min, then the collected supernatant was stored at 4 °C for further experiments. Serum AST, ALT, TG levels were used for serum biochemical analysis after administration of alcohol, Fx or Silibinin. The levels of ALT, AST, and TG in serum or liver tissue were determined according to the following commercial kit protocols obtained from Nanjing Jiancheng Bioengineering Institute (Nanjing, China).

### 3.5. Determination of Hepatic MDA and Antioxidant Enzymes

When preparing liver homogenate, 1 g of liver tissue was mixed on ice with 9 mL of normal saline and centrifuged (4000 rpm, 10 min). The protein concentration in the supernatant of liver homogenates was quantified using a BCA total protein assay kit. The hepatic level of MDA and activities of T-AOC, GSH-Px, SOD and CAT were measured by following commercial kit protocols.

### 3.6. Determination of Hepatic ADH and ALDH

A 10% liver homogenate was obtained as described in Section 3.5. The activities of hepatic ADH and ALDH were measured according to the commercial assay kits.

### 3.7. Measurement of Pro-Inflammatory Cytokines in Liver

A 10% liver homogenate was obtained as described in Section 3.5, using phosphate-buffered saline (PBS) instead of normal saline. The levels of IL-1β, IL-6, TNF-α and IFN-γ were quantified using enzyme-linked immunosorbent assay (ELISA) kits according to the manufacturer’s instructions. 

### 3.8. Western Blot Analysis 

Liver tissue stored at −80 °C was quickly placed in a mortar and liquid nitrogen was added, followed by pulverization of the material to obtain tissue powder. The powder was collected in a microcentrifuge tube for use in immunoblot analysis. The procedure for Western blotting was consistent with the method described by Tang et al. [59]. Experiments were performed using liver tissue samples with a protein concentration of 50 µg. Immunoreactive bands were colored with enhanced chemiluminescence (ECL, TransGen Biotech, Beijing, China), imaged using a FluorChem FC3 system (ProteinSimple, Waltham, MA, USA), and protein expression were quantified using Image Lab software. β-actin probed with antibody (Cincinnati, OH, USA) was used as a control.

### 3.9. Statistical Analysis 

All experiments were repeated at least three times. Data are expressed as the mean ± standard deviation (SD) (*n* = 8) and were analyzed by analysis of variance (ANOVA) using SPSS 19.0 software (IBM SPSS Statistics, Ehningen, Germany). The statistical difference was considered to be significant at *p* < 0.05.

## 4. Conclusions

In summary, our research indicates that Fx is an effective substance to prevent alcoholic liver injury. This study demonstrates that Fx attenuates alcohol-induced oxidative stress by lowering the concentration of oxidative products and up-regulating Nrf2-mediated antioxidant responses. In addition, Fx also prevents liver inflammation by inhibiting TLR4-induced signaling pathways. These findings indicate that Fx has broad prospects for the development of health foods that protect against alcoholic liver injury.

## Figures and Tables

**Table 1:** Effects of Fx on Hepatic levels of MDA and Antioxidant Enzymes in mice.

Group	T-AOC (U/mg prot)	MDA (nmol/mg prot)	SOD (U/mg prot)	GSH-Px (U/mg prot)	CAT (U/mg prot)
Control	5.72 ± 0.05 ^##^	3.07 ± 0.21 ^##^	168.52 ± 8.73 ^##^	129.57 ± 5.03 ^##^	89.61 ± 2.13 ^##^
Model	1.32 ± 0.12 **	5.90 ± 0.46 **	88.05 ± 14.71 **	69.60 ± 4.41 **	64.11 ± 2.54 **
Positive	9.34 ± 0.52 ** ^##^	3.20 ± 0.22 ^##^	156.57 ± 3.81 ^##^	129.28 ± 6.87 ^##^	96.93 ± 2.14 * ^##^
L-Fx	3.39 ± 0.17 ** ^##^	4.14 ± 0.22 ** ^##^	121.36 ± 6.23 ** ^#^	86.69 ± 3.43 ** ^##^	85.60 ± 2.09 ^##^
M-Fx	5.22 ± 0.23 * ^##^	3.79 ± 0.30 ^##^	152.34 ± 8.24 ^##^	100.75 ± 6.89 ** ^##^	105.41 ± 1.29 ** ^##^
H-Fx	9.64 ± 0.14 ** ^##^	2.91 ± 0.26 ^##^	175.74 ± 12.68 ^##^	128.79 ± 7.73 ^##^	134.00 ± 1.36 ** ^##^

Data are given as mean ± SD (*n* = 8). * *p* < 0.05, ** *p* < 0.01 vs. Control group, ^#^ *p* < 0.05, ^##^ *p* < 0.01 vs. Model group.
